# Colon Targeted Protein Nanoparticles Loaded Suppositories : Effective against Intestinal Parasites

**DOI:** 10.34172/apb.2021.056

**Published:** 2020-07-15

**Authors:** Swati Chauhan, Neha Jain, Shilani Sharma, Shagun Mehra, Upendra Nagaich

**Affiliations:** Amity Institute of Pharmacy, Amity University, Sector-125, Noida, Uttar Pradesh-201303, India.

**Keywords:** Protein nanoparticles, Metronidazole, Factorial design, Ethanol desolvation technique, Release kinetics, Indian earthworms

## Abstract

***Purpose:*** The aim of the present investigation was to formulate protein nanoparticles (PNPs) loaded suppositories for colon targeting of metronidazole (MZ), to achieve sustain release effect.

***Methods:*** PNPs were formulated via desolvation technique by utilizing 2^3^ factorial design which results into eight formulations. The synthesized PNPs were characterized for different physicochemical and in vitro parameters *viz*. particle size, surface morphology, entrapment efficiency and zeta potential, drug- excipients compatibility studies.

***Results:*** The formulated PNPs were found to be spherical in shape and have an average size in the range of 300.7 nm to 504.8 nm. Based on the results obtained, F7 was found to be the optimized formulation that was loaded into the suppository base. Furthermore, suppositories were also characterized for several parameters like content uniformity, weight variation and liquefaction time.

***Conclusion:*** Resultant, suppositories were free from pits, fissures and cracks. The *in-vitro* release data of MZ-PNPs loaded suppositories were compared with the suppositories loaded with active ingredient only i.e. MZ. Screening against *Pheretima posthuma* was also conducted. The results of *in vitro* drug release testing proved that protein nanoparticle loaded suppositories is a better approach, compared to pure MZ loaded suppositories. Release kinetic study concluded that the formulation follows Higuchi’s equation i.e. having a biphasic release pattern. The efficiency of the formulated dosage form was evaluated using Indian earthworms, *P. posthuma*.

## Introduction

In present scenario, efforts are being made to achieve more controlled drug distribution throughout the body with lessen side-effects. Drugs which are not able to attain the desired therapeutic level are being incorporated into different carrier systems varying from micro to nano range. Proteins derived nanoparticles are bio-degradable, non-antigenic, metabolizable. Due to the defined primary structure of proteins, they can contribute covalent drug attachment. Recently albumin, legumin and gelatin are being greatly used in these preparations. Due to its biodegradable and non- toxic nature, it has become the most projecting macro-molecular carrier,^1^ these are widely used to prepare nanospheres and nanocapsules. It is a major protein which provides benefits for ease of preparation in desired sizes and presence of reactive groups (thiols, amino or carboxylic groups) ligand binding for covalent linkage. Here albumin acts as a retardant agent i.e. helps to achieve prolonged release. The advantages mentioned above gives authors the basis for using albumin for preparing nanoparticles of metronidazole (MZ). MZ is a colon targeting drug, effective against *Entamoeba histolytica* and *Giardia lamblia.*^2,3^

The pharmacokinetic profile of MZ indicates that the largest amount of active ingredient is absorbed from the upper part of the intestine, furthermore, protein may also get deteriorate at gastric pH. Thus, suppositories are chosen as dosage form to prevent drug and protein nanoparticles (PNPs) from detoriation.^4-6^ In addition, albumin is highly soluble at pH 7.4 which is one of the reasons that rectal route was chosen so that, it is facile to release the drug at the targeted area.^7^ Suppositories should have effective mucoadhesive property for their retention of drug on rectal mucosa.^8,9^

The aim of the present investigation was to fabricate MZ loaded PNPs-based suppositories and to evaluate its efficacy against parasitic infections. These MZ-PNPs suppositories were also compared with MZ loaded suppositories to evaluate its in vitro efficacy and release kinetics pattern of the fabricated formulation.

## Materials and Methods

### Materials

MZ was procured from CDH Pvt. Ltd., New Delhi, India. Albumin was purchased from Sigma Aldrich Chemicals Pvt Ltd., New Delhi, India. Ethanol, glutaraldehyde, cocoa butter was purchased from CDH Pvt. Ltd., New Delhi, India.

### Methodology

#### Statistical design

2^3^ full factorial design was used for fabrication of PNPs. This design signifies the two levels (high & low) with 3 independent factors and 2 dependent variables (particle size and entrapment efficiency). In the present work, three independent factors were used i.e. albumin concentration, glutaraldehyde concentration and stirring speed. Two-factorial levels were used and coded as -1 and +1. The factorial design and formulation table has been summarized in Table 1 and Table 2.

**Table 1:** 2^3^ Factorial design: factors and levels for protein nanoparticles formulation

**S. No**	**Factors**	**Levels**	
		**-1 (Low)**	**+1 (High)**
A	Albumin (mg)	100	150
B	Glutaraldehyde (%w/v)	0.11 ml	0.9 ml
C	Stirring speed (rpm)	6500	7500

**Table 2:** Formulation Table for development of metronidazole loaded protein nanoparticles

**Formulation code**	**Albumin (mg)**	**Glutaraldehyde (%w/v)**	**Stirring speed (rpm)**
F1	100	0.11 mL	6500
F2	100	0.9 mL	7500
F3	150	0.11 mL	6500
F4	150	0.9 mL	7500
F5	150	0.11 mL	7500
F6	100	0.9 mL	6500
F7	150	0.9 mL	6500
F8	100	0.11 mL	7500

### Preparation of MZ PNPs

PNPs were prepared by ethanol desolvation technique using albumin as a polymer/retardant agent. All ingredients were accurately weighed as specified in Table 1.

Albumin was dispersed in distilled water to form an aqueous phase while organic part was formed by dissolving MZ in ethanol. Aqueous phase was added drop-wise into organic phase. The solution was kept on continuous stirring under magnetic stirring resulting into transparent suspension. Glutaraldehyde was added for initiating cross linking procedure with continuous stirring using magnetic stirrer for 24 hours. The process was carried out at room temperature. The resulted solution was sonicated for 5 minutes^10^ and further characterized for different physicochemical and *in vitro* studies.

### Preparation of MZ-PNPs loaded suppositories and MZ suppositories

MZ suppositories were formulated using cocoa butter base. The base was melted using water bath; polysorbate 80 at concentration of 5% w/w was added followed by the addition of MZ with gentle stirring to ensure complete mixing. To this melted base, optimized formulation of MZ-PNPs (equivalent to 250 mg of MZ) were added with gentle stirring to ensure uniform mixing. Then this base was poured into the metal molds and allowed to cool at temperature of 4°C. Suppositories comprising MZ (not MZ-PNPs) were also prepared by loading MZ into cocoa butter base with uniform mixing. The rest conditions were same as discussed for MZ-PNPs loaded suppositories.

### Characterization of MZ loaded PNPs

#### Scanning electron microscopic (SEM)

SEM was used for analyzing particle shape and surface morphology. It was analyzed by scanning electron microscope manufactured by Cart Zeiss EV018. The samples were prepared by lightly sprinkling nanoparticles on double-sided adhesive tape on an aluminum stub. The stubs were then coated with gold to a thickness of 200 to 500 A˚ under an argon atmosphere using a gold sputter module in a high vacuum evaporator (Quorum SC7620).^11^

### Zeta and particle size analysis

Dynamic light scattering technique was used for Zeta analysis, using a zetasizer (3000HS Malvern Instruments, UK). Samples were prepared to ten times with distilled water to form nanoparticulate suspension. Particle size analysis was performed by Photon correlation spectroscopy.

### Drug- excipients interaction studies

For drug excipients interaction studies, Fourier transform infrared spectroscopy (FTIR) was performed. The sample was mixed with dry potassium bromide powder. Pellets were prepared and kept under pressure to obtain the spectrum. Interaction studies were carried out by taking the individual spectra of pure drug (MZ), excipients (albumin) and final prepared PNPs.

### Determination of drug entrapment efficiency

All the preparations were subjected to centrifugation and the supernatants obtained was analyzed for the drug using UV spectrophotometer^12,13^ at 340 nm. Entrapment efficiency (EE) is calculated by applying the following formula:

*EE% = (Initial drug –Drug in the supernatants) / (Initial drug) ×100*

### In vitro drug release studies

These studies were performed in phosphate buffer saline (PBS) pH 7.4. Two milliliters of prepared PNPs was enclosed in dialysis bag 70 (Hi-Media, Mumbai, India) with both ends tied. These bags were kept in the PBS at 37ºC ± 0.5ºC with stirring speed of 50 rpm. At fixed period, 5 mL of the samples were taken and replaced by the same volume of freshly prepared PBS (pH 7.4). The samples were withdrawn at regular interval of time with replacement of fresh buffer solution. The samples taken were analyzed by the UV spectrophotometer at 340 nm.^14^

### Evaluation parameters for MZ PNPs loaded suppositories

#### Physical analysis

Physical analysis for suppositories was performed; it includes visual examination (color, surface condition and shape), odor and weight.^15^

### Weight variation and Mechanical strength determination

The weight variation test was evaluated with reference to the method described in British Pharmacopeia. Average weight 20 suppositories were calculated by weighing them individually. No suppositories should deviate from mean weight by more than 5% except two, which may deviate by not more than 7.5%.^16^ Mechanical strength was measured by using Monsanto hardness tester.^17^

### Content uniformity

For the evaluation of content uniformity, suppositories were taken and dissolved in 250 ml PBS (pH 7.4). The solution was filtered and content was measured by using UV spectrophotometer at 340 nm.^18^

### Liquefaction time

The liquefaction time was evaluated by using a test tube having slender aperture on one side and wider on another side. This was immersed in water maintained at 37ºC ± 0.5ºC, as the slender end faces toward hot water. Suppository was engrossed from the top of the pipette with a glass rod rested over it. The temperature at which the glass rods just come down and reaches to the end was noted and termed as liquefaction time.^19^

### Disintegration time

The disintegration time of suppository was evaluated by USP tablet disintegration (Electro lab, ED 2L) test apparatus. Distilled water was used as a medium maintained at body temperature. Suppository was placed in the metal basket and maintained at 50 rpm. Small quantity of aliquot was withdrawn and replace with equal quantity of sample and absorbance was measured at 340 nm using UV spectrophotometer.^20^

### In vitro drug release study

Dissolution method (basket method) was used for determination of in vitro drug release. *In vitro* test was done for both type of suppositories i.e. MZ suppositories as well as MZ-PNPs suppositories. Suppository was kept in basket and placed into a container having 900 ml of PBS (pH 7.4). The basket was rotated at 50 rpm at a constant temperature i.e. 37°C ± 0.5°C. At different time period aliquots were taken and simultaneously restored by equal amount of phosphate buffer. Drug concentration was measured by using UV spectrophotometer at 340 nm.^21^

### Screening against Pheretimaposthuma

The optimized MZ-PNPs loaded suppositories were evaluated for their inhibitory activity against intestinal parasites. Indian adult earthworms (*P. posthuma*) were used for this characterization. The earthworms were collected directly from moist soil and washed with water, so as to clear out dirt and other fecal matter. These removed soil and fecal matter were kept for further use to maintain artificial physiological and anatomical resemblance, thus to create and provide the arena of intestinal roundworm parasite (*Ascaris lumbricoides*) present in human beings. For screening earthworm were released into Petri plates (containing previously preserved soil and fecal matter obtained while cleaning the earthworms) along with formulated suppositories in solution form. Time taken for paralysis and death of earthworms were observed. Paralysis time was based on earthworm’s behavior with no movements when were shaken vigorously. The time of death was calculated with no movements when dipped in water (50-60°C temperature), following with faded body color of the earthworms.

## Results and Discussion

The formulated PNPs were evaluated for several physicochemical properties viz. particle size, surface morphology, zeta potential, *in vitro*drug release study. The optimized formulation of PNPs was loaded to a suppositories base and evaluated for weight variation, disintegration, liquefaction time and *in vitro*release study.

### SEM

**Figure 1:**
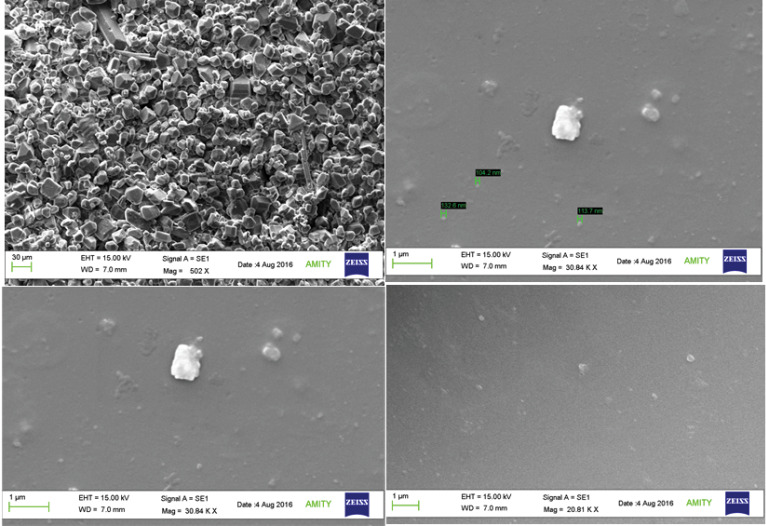
SEM images showing morphology and size of prepared protein nanoparticles

SEM concluded the particles to be spherical in shape, as shown in Figure 1and the particle size for all the formulation was found to be below 300 nm. The particles were found to have uniform size distribution and were not conglomerated.

### Determination of particle size and zeta potential measurement

The particle size was in the range of 300.7 nm to 504.8 nm. Out of all the formulation F4 was concluded as the optimized formulation, having the smallest size and optimum poly dispersity index (PDI) of 300.7 nm and 0.190 respectively. The results have been depicted in Table 3. The result obtained from particle size analysis suggested that increment of stirring speed tends to decrease the average particle size. But further increment results into increase in the particle size with the special mention to the formulation with high cross-linking agent. As, it has been suggested that as the stirring speed increases, kinetic energy also increases, resulting in variable surface charges which leads for the formation of agglomerates.^22^ Small sized nanoparticles are effective for targeted drug delivery, which allows the drug for efficient uptake by a variety of cell types and drug accumulation at target site. The PDI value was found to be 0.190 implying a narrow and favorable particle size distribution (PDI < 0.5). Zeta potential of the optimized formulation was found to be -29.2 mV. Particles having zeta potential in the range of +40 mV to -40 mV are considered to be stable. The result signifies the stability of PNPs with respect to surface charge.^22^

**Table 3:** Results showing particle size, PDI and entrapment efficiency percentage

**Batch**	**Particle Size (nm)**	**PDI**	**Entrapment efficiency (%)**
F1	465.2	2.62	58.76±0.30
F2	437.0	2.10	61.43±1.30
F3	504.8	1.83	66.23±0.61
F4	300.7	0.190	85.73±0.56
F5	348.9	0.43	70.76±0.61
F6	328.5	0.31	72.83±0.20
F7	365.6	0.93	93.10±0.30
F8	450.9	1.97	59.90±0.20

### Drug excipients interaction studies

**Figure 2:**
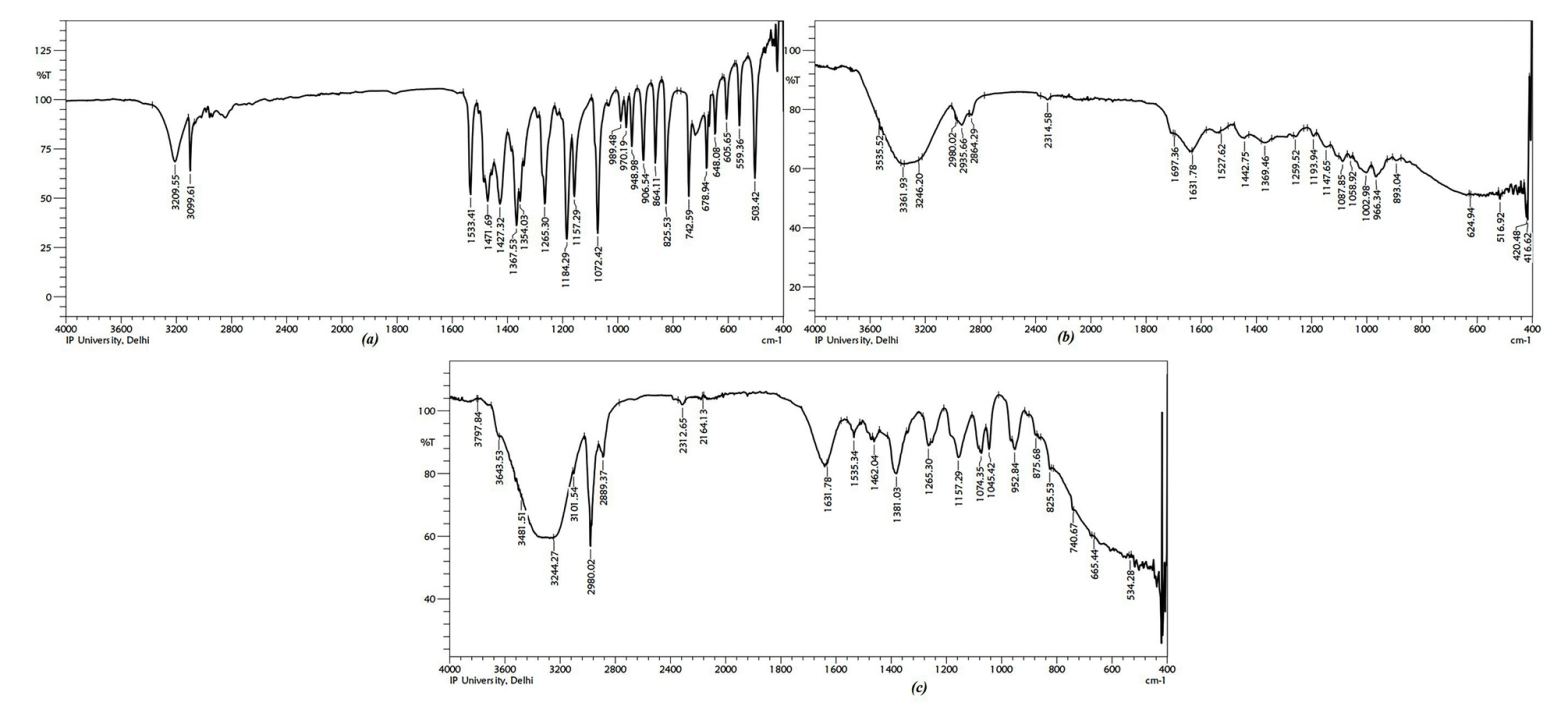
IR Spectra of (a) Metronidazole (b) Albumin (c) Formulation containing protein nanoparticles

No interaction was detected during FTIR studies between MZ and polymers used. IR spectra of pure drug i.e. MZ, albumin and the mixture of these compounds has been illustrated in Figure 2.

### Drug entrapment efficiency

EE% of all the formulations have been described in Table 3. On the basis of results obtained, it can be concluded that F7 was having the highest entrapment efficiency i.e. 93.10±0.30%. It was observed that percent entrapment efficiency varies with change in stirring speed and concentration of cross-linking agent. From the results it can be concluded that increase in the stirring speed leads to decrease the particle size, thus reducing the percentage drug entrapment efficiency but gets increased on increasing the cross-linking agent concentration.^23-25^

### In vitro drug release study of MZ-PNPs

**Figure 3:**
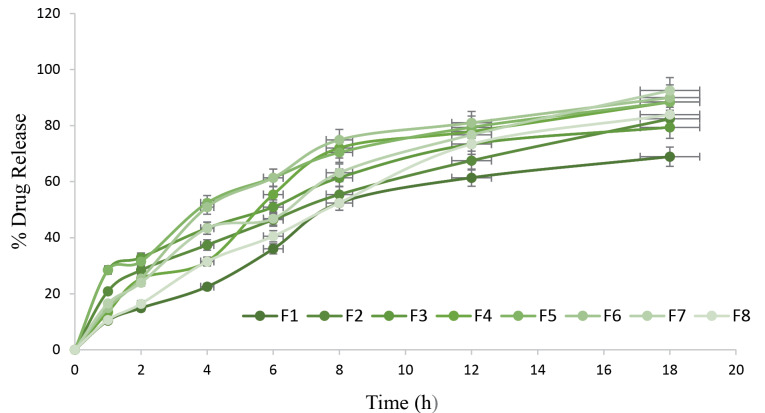
In vitro drug release graphs for metronidazole loaded protein nanoparticles

*In vitro* drug release for all the formulation have been depicted in Figure 3. From the graph it was observed that F4 and F7 attains the highest drug release for the time period of 18h. Based on the outcomes, it can be concluded that due to small size of particles, sustain release effect was observed during *in vitro* release study.

### Characterization of MZ-PNPs loaded suppositories

The formulated MZ-PNPs suppositories were free from any cracks and fissures. Suppositories were evaluated on the basis of visual examination; the results have been summarized in Table 4. The suppositories were found to have Ogive shaped with smooth texture; having yellowish white in color.

**Table 4:** Compiled results of characteristics for suppositories parameters

**Parameters**	**Observations**
Physical shape	Ogive shaped
Surface condition	Smooth
Color	Yellowish white
Odor	Odorless
Mechanical strength (kg/cm^3^)	1.8±0.97
Liquefaction time (min)	27±0.12
Disintegration Time (min) (37.5±0.5℃)	6.98±0.71

Readings were taken in triplicate n=3 (mean ± SD).

The weight variations for the formulated suppositories were found to be in acceptable limit as provided in British pharmacopoeia, furthermore suppositories showed optimal mechanical strength which is a necessary part for handling and transportation purpose.^16^ The results are compiled in the Table 4.

### Weight variation

The weight variation study for all the suppositories were found to be less than 5% and according to British Pharmacopoeia this obtained percentage is acceptable, indicating that mold calibration was ideal. The results have been shown in Table 4.

### Content uniformity

The content uniformity was within the permissible range (97% to 105%) indicating uniformity of drug dispersion in suppositories. The results have been shown in Table 4.

### Mechanical strength

The formulated suppositories were found to acquire acceptable mechanical strength. The results have been compiled in Table 4.

### Liquefaction time

The liquefaction time of the prepared suppositories was found to attain the time period of 27 minutes. The results have been depicted in Table 4 and are discussed in the discussion part.

### Disintegration time

According to British Pharmacopoeia the disintegration time for suppositories should be less than 60 minutes and the results show the same. The average disintegration time was found to be 6.98 minutes depicted in Table 4.

### In vitro release of MZ PNPs - suppositories and MZ loaded suppositories

**Figure 4:**
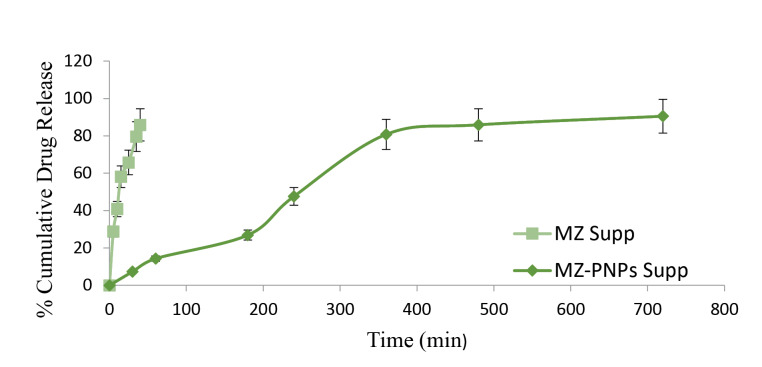
Comparative In vitro drug release of MZ suppositories and MZ-PNPs suppositories

The *in vitro* release of MZ suppositories showed that about 50% of the drug releases within 25 minutes of drug administration whereas, it took 4 hours for MZ-PNPs loaded suppositories to release its 50% of the drug. For MZ-PNPs loaded suppositories 90% of the drug releases for 12 hours defining a sustained release pattern. It was also observed that MZ-PNPs loaded suppositories gets immediately melts with body temperature and a burst release of drug release followed by sustain release was seen due to presence of PNPs. The comparative release graph of MZ loaded suppositories and MZ-PNPs loaded suppositories have been depicted in Figure 4.

**Figure 5:**
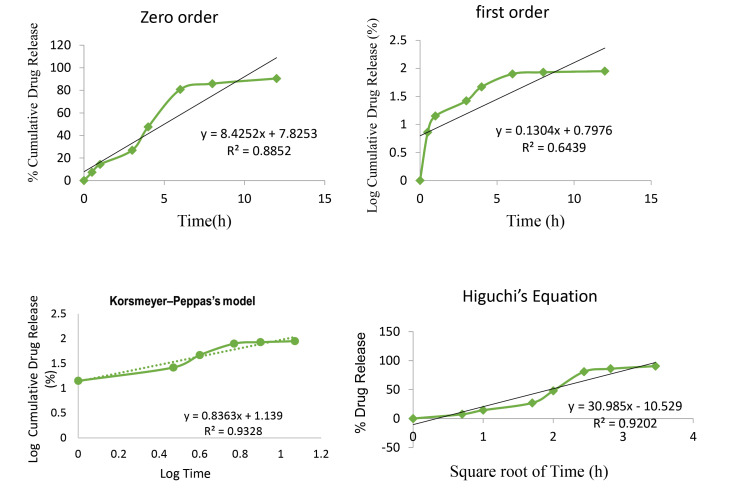
Release kinetics of MZ-PNPs loaded suppositories

For release kinetics, the model or equation that best fits the release data was evaluated by correlation coefficient (r) and the value of n (for Korsmeyer Peppas’s equation). It was found that the *in vitro* release from formulated MZ-PNPs loaded suppositories was best explained by Higuchi’s equation with the highest linearity (R^2^=0.920), followed by zero order (R^2^=0.885) and first order (R^2^=0.643), showed in Figure 5. The value of n=1.139 (greater than 0.45) obtained from Korsmeyer Peppas’s equation shows that the model follows anomalous (non-Fickian) diffusion process. The release kinetics helps the authors to indicate the Higuchi’s equation, as the best suited model, demonstrating a biphasic release pattern having an initial burst release followed by a slow sustained release pattern.^26,27^

### Screening against Pheretimaposthuma

Screening of MZ-PNPs loaded suppositories against intestinal parasiteswas conducted on Indian adult earthworm (*P. posthuma*) because of its anatomical and physiological resemblance with the intestinal roundworm parasites of human beings. The result obtained showed that the formulated dosage form is able to act against these earthworms. The analyzation of paralysis time was based on earthworms’ behavior with no movements while shaken vigorously. Formulated suppositories in the solution form take 6.50±0.05 minutes for death of earthworms, while 3.47± 0.05 minuteswere taken for paralysis of earthworms. The results obtained showed its effectivity against *P. posthuma*.

## Conclusion

The ability MZ-PNPs loaded suppositories was demonstrated by the results obtained from various evaluation parameters viz. particle size, poly dispersity index, entrapment efficiency and in vitro release profile. The in vitro release study confirmed the prolongation of the drug release time. It can be concluded that the effective and safe dosage form was prepared with reduced side effects for inhibiting parasitic infections. The prepared MZ-PNPs suppositories provide an effective targeted delivery of drug with the release for 12 hours. Release kinetics results showed the best fitted model (Higuchi’s equation) signifying the phenomenon of sustain release effect of fabricated formulation.

## Ethical Issues

Not applicable.

## Conflict of Interest

The authors declare that there is no conflict of interest regarding the publication of this paper.

## Acknowledgments

The authors wish to thank Founder President Dr. Ashok K. Chauhan, Amity University for his kind support during the research.

## References

[1] Sailaja AK, Amareshwar P, Chakravarty P (2011). Different techniques used for the preparation of nanoparticles using natural polymers and their application. Int J Pharm Pharm Sci.

[2] Gardner TB, Hill DR (2001). Treatment of giardiasis. Clin Microbiol Rev.

[3] Elzatahry AA, Eldin MSM (2008). Preparation and characterization of metronidazole-loaded chitosan nanoparticles for drug delivery application. Polym Adv Technol.

[4] Bruno BJ, Miller GD, Lim CS (2013). Basics and recent advances in peptide and protein drug delivery. Ther Deliv.

[5] Renukuntla J, Vadlapudi AD, Patel A, Boddu SH, Mitra AK (2013). Approaches for enhancing oral bioavailability of peptides and proteins. Int J Pharm.

[6] Vaidya A, Jain S, Agrawal RK, Jain SK (2015). Pectin–metronidazole prodrug bearing microspheres for colon targeting. J Saudi Chem Soc.

[7] Lohcharoenkal W, Wang L, Chen YC, Rojanasakul Y (2014). Protein nanoparticles as drug delivery carriers for cancer therapy. Biomed Res Int.

[8] Shaikh R, Raj Singh TR, Garland MJ, Woolfson AD, Donnelly RF (2011). Mucoadhesive drug delivery systems. J Pharm Bioallied Sci.

[9] Jithan A, Madhavi K, Madhavi M, Prabhakar K (2011). Preparation and characterization of albumin nanoparticles encapsulating curcumin intended for the treatment of breast cancer. Int J Pharm Investig.

[10] Nagaich U, Gulati N, Chauhan S (2016). Antioxidant and antibacterial potential of silver nanoparticles: biogenic synthesis utilizing apple extract. J Pharm (Cairo).

[11] Bhabani Shankar N, Prasant Kumar R, Udaya Kumar N, Benoy Brata B (2010). Development and characterization of bioadhesive gel of microencapsulated metronidazole for vaginal use. Iran J Pharm Res.

[12] Mukherjee B, Santra K, Pattnaik G, Ghosh S (2008). Preparation, characterization and in-vitro evaluation of sustained release protein-loaded nanoparticles based on biodegradable polymers. Int J Nanomedicine.

[13] Oranje AP (2014). Evidence - based pharmacological treatment of atopic dermatitis: an expert opinion and new expectations. Indian J Dermatol.

[14] Shanmugam S, Kim YH, Park JH, Im HT, Sohn YT, Kim KS (2014). Sildenafil vaginal suppositories: preparation, characterization, in vitro and in vivo evaluation. Drug Dev Ind Pharm.

[15] Ghorab D, Refai H, Tag R (2011). Preparation and evaluation of fenoterol hydrobromide suppositories. Drug Discov Ther.

[16] Zidan AS, Emam SE, Shehata TM, Ghazy FE (2015). Pediatric suppositories of sulpiride solid dispersion for treatment of Tourette syndrome: in vitro and in vivo investigations. AAPS PharmSciTech.

[17] Saleem MA, Taher M, Sanaullah S, Najmuddin M, Ali J, Humaira S (2008). Formulation and evaluation of tramadol hydrochloride rectal suppositories. Indian J Pharm Sci.

[18] Sah ML, Saini TR (2008). Formulation development and release studies of indomethacin suppositories. Indian J Pharm Sci.

[19] Zawar LR, Bhandari GS (2012). Formulation and evaluation of sustained release ondansetron poloxamer based solid suppositories. J Appl Pharm Sci.

[20] Nagaich U, Chaudhary V, Tonpay SD, Karki R (2010). Design and evaluation of a metronidazole central core matrix tablet. J Adv Pharm Technol Res.

[21] Yue PF, Yuan HL, Yang M, You RH, Cong LB, Zhu J (2008). Preparation, characterization, and pharmacokinetic evaluation of puerarin submicron emulsion. PDA J Pharm Sci Technol.

[22] Awotwe-Otoo D, Zidan AS, Rahman Z, Habib MJ (2012). Evaluation of anticancer drug-loaded nanoparticle characteristics by nondestructive methodologies. AAPS PharmSciTech.

[23] Maghsoudi A, Shojaosadati SA, Vasheghani Farahani E (2008). 5-Fluorouracil-loaded BSA nanoparticles: formulation optimization and in vitro release study. AAPS PharmSciTech.

[24] Ramteke KH, Nath L (2014). Formulation, evaluation and optimization of pectin- bora rice beads for colon targeted drug delivery system. Adv Pharm Bull.

[25] Anarjan N, Jafarizadeh-Malmiri H, Nehdi IA, Sbihi HM, Al-Resayes SI, Tan CP (2015). Effects of homogenization process parameters on physicochemical properties of astaxanthin nanodispersions prepared using a solvent-diffusion technique. Int J Nanomedicine.

[26] Vandghanooni S, Eskandani M, Barar J, Omidi Y (2018). AS1411 aptamer-decorated cisplatin-loaded poly(lactic-co-glycolic acid) nanoparticles for targeted therapy of miR-21-inhibited ovarian cancer cells. Nanomedicine (Lond).

[27] Hamishehkar H, Bahadori MB, Vandghanooni S, Eskandani M, Nakhlband A, Eskandani M (2018). Preparation, characterization and anti-proliferative effects of sclareol-loaded solid lipid nanoparticles on A549 human lung epithelial cancer cells. J Drug Deliv Sci Technol.

